# Medical and Physical Intelligence

**Published:** 1804-03-01

**Authors:** 


### MEDICAL AND PHYSICAL INTELLIGENCE.

### [ FOREIGN AND DOMESTIC. ]

Our Readers have been acquainted in a former Number of this Journal, of Dr. Trommsdorf's discovery of a new earth, which he found in the Saxon beryll, and to which he gave the name of Agust Earth, on account of its forming tasteless salts. The fossil itself, in which it was said to be contained, has since been received into the system of mineralogy by the name of Agustit. This discovery however is now annihilated by the latest researches of Hauy, Vauquelin, Klaproth, and Karsten, and the agust earth is to be exploded from the catalogue of simple bodies. Mr. Karsten, of Berlin, who was one of the first mineralogists that admitted the agustit in his valuable mineralogical tables, at the request of Mr. Vauquelin, transmitted to him a small quantity of this fossil, adding also some specimens for Mr. Hauy, as both gentlemen were curious in convincing themselves of the identity of the fossil, as well as of the new earth contained in it. In a letter which Mr. Karsten received some time after from Mr. Hauy, he was informed of the arrival of his present, and, at the same time, of the interesting results which Vauquelin and Hauy had obtained by their analysis of the Saxon beryll. Mr. Hauy examinining this fossil according to his method, found it to have the same form as the phosphat of lime or apatit, and the chemical analysis of Vauquelin agreed exactly with the opinion of his colleague; for he could trace nothing in it but phosphat of lime. Mr. Karsten, surprised at receiving this intelligence gence from those great naturalists, re-examined a small quantity of one part of that fossil, of which he had sent the other part to Paris; and the few experiments he could at that time undertake, seemed to yield the same results with those of Hauy and Vauquelin; but in order to obtain a perfect chemical analysis of the agustit, he requested Mr. Klaproth to submit it to a new analysis, acquainting him at the same time with the intelligence received from Paris. This gentleman immediately complied with his request, and sent him soon after the following account: "Pure pieces of augustit, after having been reduced to a fine powder, were infused with nitric acid, in which the powder dissolved for the most part; but on heating the liquor a perfect solution was effected. The clear liquor was divided into two parts. 1. One half of it was accurately neutralised with ammonia and treated with a solution of quicksilver in nitric acid, which had been prepared in the cold. A phosphat of mercury was precipitated, which being properly heated, left behind phosphoric acid after the evaporation of the mercury. 2. The rest, or the second half of the above-mentioned solution, on being treated with oxalate of kali, yielded oxalate of lime. This substance, edulcorated, dried, and strongly heated in a platina crucible, gave quick lime, which being infused with water formed lime water; and dissolved in nitric acid and precipitated by sulphuric acid, it gave sulphat of lime or gypsum; and precipitated by carbonated kali, it was changed into carbonat of lime." From these experiments it appears, that what is generally called Saxonian beryll is only a variety of apatit; and that if Mr. Trommsdorf has not analysed a different fossil, which he might have received by the false name of Saxon beryll, the existence of the agust earth must be denied, till further information is received from that gentleman.

---

Dr. Friese, of Breslau, has employed the digitalis in three cases of pulmonary consumption with the greatest success, and thus added a new evidence of the antiphthisical virtue of that efficacious plant. The patients were in the first stage of the disease, and two of them affected with tubercles. They spat blood, discharged a great quantity of purulent matter, and had a high degree of fever, and colliquative sweats. The remedy was given in form of powder and in infusion, and the cure was concluded with bark and lichen islandicus. The digitalis was given by degrees in such doses till its narcotic effect could be perceived; it generally caused vomiting, giddiness, and dilatation of the pupil. It visibly diminished the number of pulsations; and in one case the pulse fell, in the course of six days from 110 and 115 to 60 beats in a minute, and was raised again to more than 80, on having discontinued the remedy for some days.

---

Cit. Conte has discovered a new mode of preventing the oxydation or rusting of iron and steel. For this purpose he recommends a coma composition of fat oil varnish, with the same quantity or at least with four-fifths of rectified oleum terebinthinæ. This varnish is laid on thin and even by means of a sponge, and is suffered to dry in a place where it cannot be spoiled by dust. It preserves the metallic gloss, and no trace of rust is ever perceived on things thus varnished. It may likewise be applied on copper, the polish and colour of which are always preserved by it, and it is particularly recommendable for surgical and other instruments.

---

Mr. Renaud is of opinion, from some experiments recently made, that arsenic in a metallic state has no effect on the animal body. Metals, in general, seem only to act on the animal body, when in a state of oxydation; hence all metals which are not easily oxydable, have but little or no effect on the living body. Thus quicksilver, as a metal, has not the least action, but only a mechanical one, depending on its natural heaviness; whereas, in an oxydated state it proves strongly stimulating, and often corrosive. Many difficulties however occur in making experiments with metallic arsenic, this metal being already oxydable by the mere contact of the air; besides, the slightest trituration is capable of oxydating it, on which account it cannot be given in powder; and in pieces it will only act with its surface, because it dissolves with great difficulty in the stomach. For these reasons, Mr. R. took the arsenial mundic (mispickel in German) which may be reduced to a fine powder without losing its metallic gloss. Mr. R. gave this substance from 18 gr. to 2 drachms to dogs, without perceiving any symptoms of a deleterious effect.

---

Signor Brugnatelli has communicated, in a letter to Van Mons, the results of several experiments with the action of Volta's pile on animal fluids. Blood taken from an ox, lost its colour and cogulated, when brought in contact with the positive metallic plate; at the negative pole it received only a black colour. Milk brought in connection with the positive pole, coagulated, receiving an agreeable acid taste; the negative pole was covered with saccharum lactis. On repeating the experiment with gold wire instead of platina, the coagulated milk received a rose colour. Saliva slightly coagulated at the positive pole; but ox-gall coagulated at this pole to a substance exactly resembling that which is produced by treating the gall with acid. Urine deposited urée at the positive pole, and at the negative, phosphat of ammonia.— The white of an egg coagulated at the positive pole, in the same manner as by the application of heat; and the yolk of an egg was hardened round the metallic plate.

---

The largest specimen of amber, which has ever been discovered, was accidentally found in a meadow near Insterburg, in Prussia. It

It is almost as long as half a sheet of common paper, and exactly as broad, and about two inches and a half thick; its weight is thirteen pounds, seven ounces and a half. It is pale yellow, and its surface covered with a brown opaque crust. Its value however is much lessened by several holes that penetrate toward the middle. It is said that 5000 dollars, or about 900l. have been offered for it; and the Armenian merchants, who trade in amber, have asserted, that it might probably be sold for 8000l. in Constantinople. It is now preserved in the Royal Chamber at Koenigsberg.

---

Mr. BRUGNATELLI observes, that on treating common paper with nitric acid, we obtain a great quantity of suberic acid mixed with oxalic acid, which proved that M. Fourcroy was right in ranking cork amongst the immediate constituents of vegetables.

---

M. CHENEVIX, in a letter, dated Dresden, observes, that Des Costils dissolves raw platina in nitro-muriatic acid, and precipitates it by muriate of ammonia at several times. The first portions are yellow, the last redder. He reduces the red precipitate, and obtains an alloy. He exposes this alloy to a current of oxygen, and a blue oxyd is volatilized; pure platina remains behind. The blue sublimate is the oxyd of this new metal.

---

A new vegetable salt, containing a new acid, has been discovered by KLAPROTH. It consists of a saline mass, exuded from the trunk of the white mulberry, *morus alba*, *L.* on the surface of the bark; it has the appearance of a coating, in little granulous drops of a yellowish and blackish brown.

---

Mr. WHATELY has in the press a work on an Improved Method of treating Strictures in the Urethra.

---

Mr. SYER will commence his Third Course of Lectures on the Elements and Practice of Midwifery, &c. at his Lecture Room, No. 102, Leadenhall Street, on Thursday, March 1, 1804, at half an hour past five o'clock in the evening.

